# Characterization of the Heat-Stable Proteome during Seed Germination in Arabidopsis with Special Focus on LEA Proteins

**DOI:** 10.3390/ijms22158172

**Published:** 2021-07-29

**Authors:** Orarat Ginsawaeng, Michal Gorka, Alexander Erban, Carolin Heise, Franziska Brueckner, Rainer Hoefgen, Joachim Kopka, Aleksandra Skirycz, Dirk K. Hincha, Ellen Zuther

**Affiliations:** 1 Max Planck Institute of Molecular Plant Physiology, Am Muehlenberg 1, 14476 Potsdam, Germany; ginsawaeng@mpimp-golm.mpg.de (O.G.); michal.gorka@celonpharma.com (M.G.); Erban@mpimp-golm.mpg.de (A.E.); carolin.heise@uni-rostock.de (C.H.); brueckner@mpimp-golm.mpg.de (F.B.); hoefgen@mpimp-golm.mpg.de (R.H.); kopka@mpimp-golm.mpg.de (J.K.); skirycz@mpimp-golm.mpg.de (A.S.); hincha@mpimp-golm.mpg.de (D.K.H.); 2 Celon Pharma S.A., Marymoncka 15, Kazun Nowy, 05-152 Czosnow, Poland; 3 Institute of Life Science, University of Rostock, Albert-Einstein-Str. 3, 18059 Rostock, Germany; 4 Boyce Thompson Institute, Cornell University, Ithaca, NY 14853, USA

**Keywords:** late embryogenesis abundant, Arabidopsis, seed germination, metabolomics, heat-stable proteome, dehydrins, LEA transcripts, intrinsically disordered proteins

## Abstract

During seed germination, desiccation tolerance is lost in the radicle with progressing radicle protrusion and seedling establishment. This process is accompanied by comprehensive changes in the metabolome and proteome. Germination of Arabidopsis seeds was investigated over 72 h with special focus on the heat-stable proteome including late embryogenesis abundant (LEA) proteins together with changes in primary metabolites. Six metabolites in dry seeds known to be important for seed longevity decreased during germination and seedling establishment, while all other metabolites increased simultaneously with activation of growth and development. Thermo-stable proteins were associated with a multitude of biological processes. In the heat-stable proteome, a relatively similar proportion of fully ordered and fully intrinsically disordered proteins (IDP) was discovered. Highly disordered proteins were found to be associated with functional categories development, protein, RNA and stress. As expected, the majority of LEA proteins decreased during germination and seedling establishment. However, four germination-specific dehydrins were identified, not present in dry seeds. A network analysis of proteins, metabolites and amino acids generated during the course of germination revealed a highly connected LEA protein network.

## 1. Introduction

Seeds are the desiccation-tolerant life stage of most plant species. However, seedlings lose their desiccation tolerance during the early stages of germination [1,2,3]. In the vegetative stage, with the exception of a small number of resurrection plants, the vast majority of species are desiccation-sensitive (see [4] for a recent review).

During germination, the quiescent embryo resumes growth and development, resulting in seedling establishment under favorable environmental conditions. The process of germination is initiated upon imbibition and has been divided into three phases [5,6]. Phase I consists of a rapid uptake of water which allows the initiation of metabolic activity, such as protein synthesis from stored mRNAs. During Phase II, further water uptake is limited. Phenotypically, testa and endosperm rupture are observed, followed by radicle protrusion, which marks the end of this phase. Gene transcription from newly synthesized mRNAs and protein synthesis results in further activation of metabolism. In Phase III, also referred to as the post-germination phase, water uptake increases again and seedling development is observed. This includes cell division, elongation of the radicle and mobilization of stored reserves to fuel seedling development before plants become photoautotrophic. After emergence of the radicle, seedling establishment progresses by emergence of hypocotyl and cotyledons and a new growth stage begins with the full opening of the cotyledons [7].

This complex developmental process has been investigated on metabolic [8,9,10,11], transcriptomic [10,12,13,14,15,16,17] and proteomic [18,19,20,21,22,23,24,25,26] levels. In all cases, distinct regulatory patterns have been observed for different metabolites, transcripts and proteins during germination and seedling development in different species. Among the metabolites that are typically found in high concentrations in dry seeds are sucrose and raffinose-family oligosaccharides (RFO) such as raffinose, stachyose and verbascose (see [27] for review). These sugars are mainly responsible for the formation of a cytosolic glass phase during maturation drying, which protects the cells from damage as water content declines [28,29,30]. Obviously, this process needs to be reversed during germination to allow full metabolic activity.

In addition to seed storage proteins, late embryogenesis abundant (LEA) proteins are highly abundant in dry seeds [31]. They were first discovered in cotton seeds during the late stages of seed development [32,33,34]. Besides seeds, they are also found in vegetative plant organs, mainly in response to dehydration stress. For example, it has been shown that LEA proteins can protect enzymes and membranes during freezing and drying [35,36,37,38]. LEA proteins are also found in other organisms including algae, anhydrobiotic invertebrates and bacteria [35,37,39]. In plants, LEA proteins have been linked to the desiccation tolerance of seeds as their accumulation coincides with the development of desiccation tolerance in orthodox seeds and their accumulation is decreasing in germinating seeds when they become desiccation sensitive [18,40,41]. In Arabidopsis, the transcripts encoding several LEA proteins have also been detected in dry, mature seeds [42]. However, the role of specific LEA proteins during seed germination is not known yet. A detailed temporal proteome analysis during seed germination and seedling establishment will help shed light on their importance during these processes.

Most LEA proteins contain a high fraction of hydrophilic amino acid residues and have been computationally and experimentally classified as intrinsically disordered proteins (IDPs) [38,42,43,44,45]. This lack of folding in solution and the low fraction of hydrophobic amino acids make most LEA proteins heat-stable [35,36,37,38]. This property has been used, on the one hand, for the purification of recombinant LEA proteins (see, e.g., [45,46] for reviews), and on the other hand, to enrich LEA proteins from seed extracts of *Medicago truncatula* in a heat-stable proteome [41,47]. In *Arabidopsis thaliana*, a total of 51 LEA proteins across nine different groups based on Pfam family domains have been identified, of which 34 were classified as seed-expressed based on the presence of the corresponding mRNAs in dry seeds [42].

Here, we explored the heat-stable proteome in dry and germinating seeds and young seedlings of Arabidopsis with a special focus on low abundant proteins. The assignment of heat-stable proteins to functional metabolic categories was investigated. LEA proteins and the abundance of LEA transcripts was analyzed using proteomics and qRT-PCR approaches, respectively. In addition, we investigated the dynamics of the seed metabolome for comparison with previous publications to improve our understanding of the complex metabolic changes taking place during germination and seedling establishment.

## 2. Results

To investigate the early stages of germination in Arabidopsis, seeds were imbibed and then subsequently collected at different time points. We collected dry seeds (DS) and germinating seeds and seedlings (Supplemental Figure S1). All treatments, except DS, also experienced a stratification period, which might have an additional impact on the results. After imbibition, we observed testa rupture (24 HAI; hours after imbibition), elongation of radicles (36 HAI), greening of cotyledons (48 HAI), hypocotyl and cotyledon emergence (60 HAI) and fully opened cotyledons (72 HAI).

### 2.1. Dynamics of Seed Metabolites and Amino Acids after Imbibition

As metabolic levels during seed germination of Arabidopsis were previously reported [8,10,11], we firstly characterized the molecular status during imbibition on the metabolic level for potential comparisons and additional evaluation of the complex changes.

Small hydrophilic metabolites were analyzed using gas chromatography-mass spectrometry analysis. After excluding known contaminants and potential contaminants identified by hierarchal cluster analysis, the data set contained 100 metabolites (see Supplemental Table S1 for the complete data set). More than half of the metabolites detected in all time points increased in abundance during germination and seedling establishment. Six metabolites (A148003, A225007, erythronic acid, mannitol, uracil and uric acid) were only found in DS.

**Figure 1:**
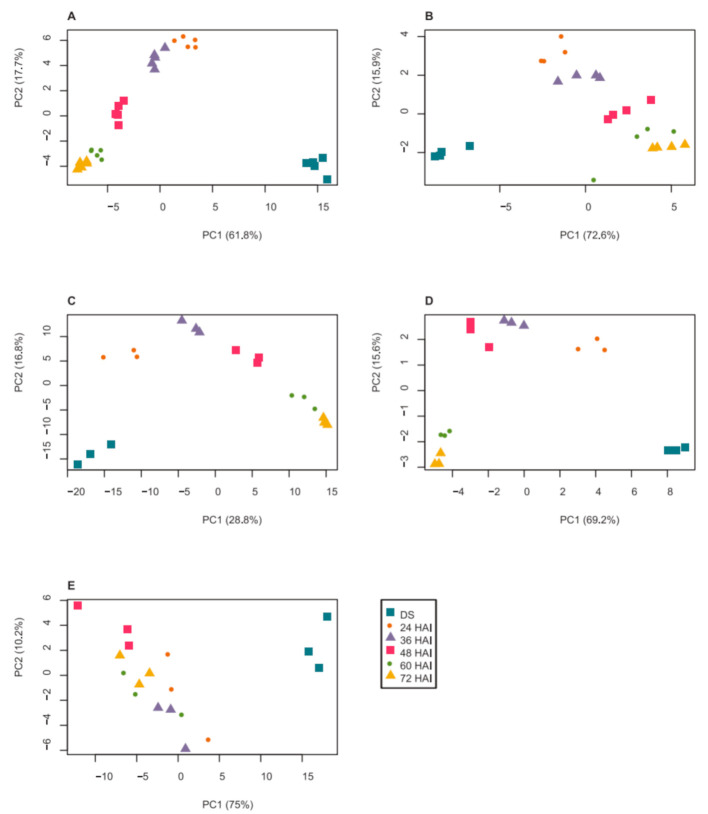
PCA of (**A**) metabolites, (**B**) amino acid composition, (**C**) heat-stable proteome, (**D**) LEA proteins and (**E**) expression of genes encoding LEA proteins of dry seeds (DS), germinating seeds and seedlings at different hours after imbibition (HAI). Data are log_2_-transformed, median-normalized mass spectral intensities (*n* = 5) (**A**), concentration of amino acid (nmol/gDW) after log_2_ transformation and median normalization (*n* = 4) (**B**), mass spectral label-free quantification (LFQ) intensities after log_2_ transformation and median normalization (*n* = 3) (**C**,**D**) or DW-normalized absolute transcript copy number in log_2_ with median normalization (*n* = 3) (**E**). Missing values were replaced with 0 prior to the analysis. Samples at different HAI are as indicated by the legend.

Principal component analysis (PCA) showed a clear separation between the metabolite composition of DS and all other time points along principal component 1 (PC1), which explained 61.8% of the total variance in the data set (Figure 1A). The different time points after imbibition were mainly separated by PC2, explaining 17.7% of the total variance, indicating that imbibition had the most drastic effect on metabolite composition, compared to further germination. Data obtained from some data points clustered more closely together, suggesting that the metabolite composition was similar in testa ruptured and radicle protruding seeds (24 and 36 HAI) and also before and after cotyledon opening in seedlings (60 and 72 HAI).

**Figure 2:**
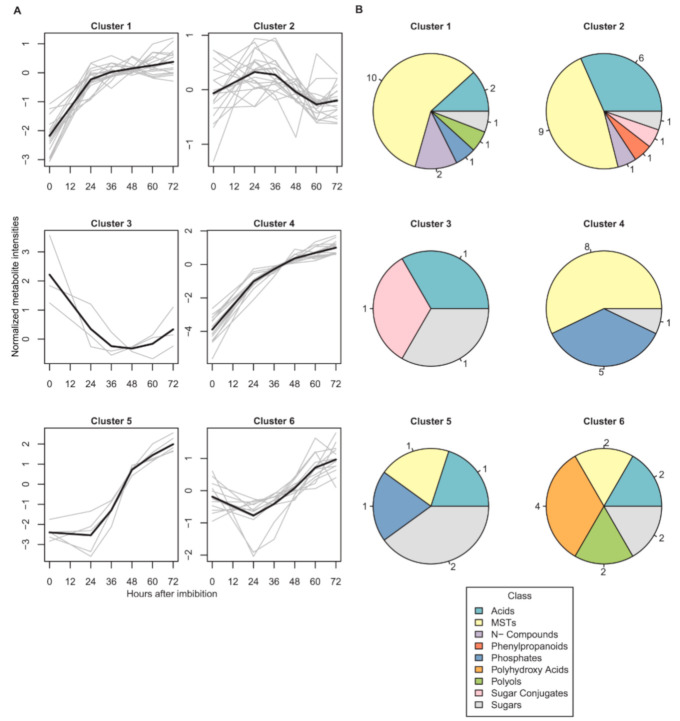
Patterns of metabolite abundance as determined by k-means clustering (clusters 1–6) (see Supplemental Figure S2) (**A**). Gray lines represent individual metabolites while black lines show the mean of each cluster. (**B**) Distribution of metabolite classes in each cluster. Amino acids were considered separately. Each chemical class is represented by a unique color as shown (MSTs = mass-spectral tags). Numbers around the pie charts represent the numbers of class members.

To resolve the different kinetic patterns of the metabolites (Supplemental Figure S2), we subjected the data of those that were detected at all time points (70% of the data set) to k-means clustering (Figure 2A). We observed six clearly resolved kinetic patterns, four of which showed an overall increase in metabolite content over 72 HAI. Cluster 1 and cluster 4 represented metabolites with a gradual increase over time from DS to 72 HAI with a higher increase in cluster 4. Similarly, metabolites in cluster 5 were highly increased from 24 HAI onwards. Cluster 6 was characterized by a higher abundance of the respective metabolites already in DS and more gradual changes over 72 HAI. The largest cluster 2 represented metabolites with only small fluctuations, especially at early time points after imbibition. The smallest cluster 3, contained metabolites with a high abundance in dry seeds and a steep decrease after 24 HAI or 36 HAI.

Metabolites of the six clusters were assigned to nine metabolite classes (Figure 2B). The most diverse clusters were cluster 1 (acids, unknowns (MSTs; mass spectral tags), N-compounds, phosphates, polyols and sugars) and cluster 2 (acids, MSTs, N-compounds, phenylpropanoids, sugar conjugates and sugars). Sugars was the only class found in all clusters, with the highest proportion in cluster 5 (40%). Acids were detected in almost every cluster except for cluster 4. MSTs, found in all clusters except for cluster 3, was the class with the highest number of members in the clustered data set (42.9%). Phenylpropanoids were exclusively observed in cluster 2.

**Figure 3:**
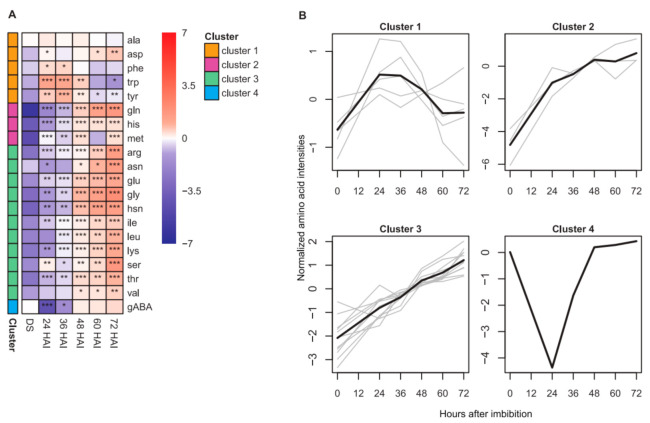
Amino acid levels in dry seeds (DS), germinating seeds and seedlings and their kinetic patterns. (**A**) Abundance of amino acids in DS, germinating seeds and seedlings collected at the indicated time points after imbibition. The color gradient represents mean concentration of amino acid (nmol/gDW) after log_2_ transformation and median normalization (*n* = 4). Levels of significance comparing the amino acids in germination seeds and seedlings to DS (unpaired two-sided *t*-test) are indicated by asterisks (* *p* < 0.05; ** *p* < 0.01; *** *p* < 0.001). Amino acids are listed alphabetically within their clusters (cluster 1–4) as determined by k-means clustering. Each cluster is indicated by a unique color. (**B**) Patterns of amino acid abundance as determined by k-means clustering. Gray lines represent individual metabolites while black lines show the mean of each cluster. gABA–γ-aminobutyric acid.

The abundance of 20 amino acids in DS, germinating seeds and seedlings was measured using reverse High-Performance Liquid Chromatography (HPLC) (Supplemental Table S2). A PCA plot of amino acid concentrations (Figure 1B) showed a similar pattern to the PCA of metabolites. A separation of DS samples from other samples collected 24–72 HAI could be observed by PC1, which explained 72.6% of the total variance. Mainly PC2, explaining 15.9% of the total variance, separated samples collected after seed germination. The changes in amino acids during seed germination clustered into four groups according to their kinetic patterns (Figure 3). It revealed that most amino acids (75%) increased in abundance over time from DS to seedlings (Figure 3A). Amino acids with increased abundance clustered into cluster 2 and also cluster 3 with the largest number of members (55%) and a more pronounced increase (Figure 3B). The abundance of amino acids in cluster 1 slightly fluctuated during DS-to-seedling transition. Lastly, the abundance of γ-aminobutyric acid (gABA) as the only member of cluster 4 decreased drastically 24 HAI and recovered 48 HAI.

### 2.2. Changes in the Heat-Stable Proteome during Germination and Seedling Development

The strong domination of seed storage proteins in dry seeds [48] makes it difficult to detect lower abundant proteins, e.g., LEA proteins. Thus, the heat-stable proteome was characterized for the detection of lower abundant proteins, especially chaperones, which may be important for seed germination and seedling establishment. We used a heat treatment to remove seed storage proteins and other heat-sensitive proteins before analyzing the composition of the remaining heat-stable proteome in seeds before and after imbibition by Liquid Chromatography-Mass Spectrometry (LC-MS/MS) analysis of proteolytic peptides. This proteomic approach revealed the presence of 1545 different proteins. After removing known contaminants, proteins represented by only one unique peptide and proteins with missing values in at least two of the three replicates in all time points, 898 proteins remained in the data set (see Supplemental Table S3 for the complete data set). Not all proteins of the heat-stable proteome were detectable at every time point—72.6%, 45.3%, 31.3%, 22.3%, 28.4% and 24.3% of the total number of proteins were missing from the samples collected at DS, 24, 36, 48, 60 and 72 HAI, respectively.

PCA revealed a similar separation of the heat-stable proteins from different time points after imbibition (Figure 1C) as observed for amino acids (Figure 1B). PC1 separated DS from germinating seeds and seedlings, explaining 28.8% of the total variance in the proteomic data set, while PC2 (16.8% of the total variance) separated samples at different time points after imbibition. Data obtained from seedlings 60 and 72 HAI clustered closely together, suggesting that protein composition remained similar in seedlings once cotyledons had emerged.

**Figure 4:**
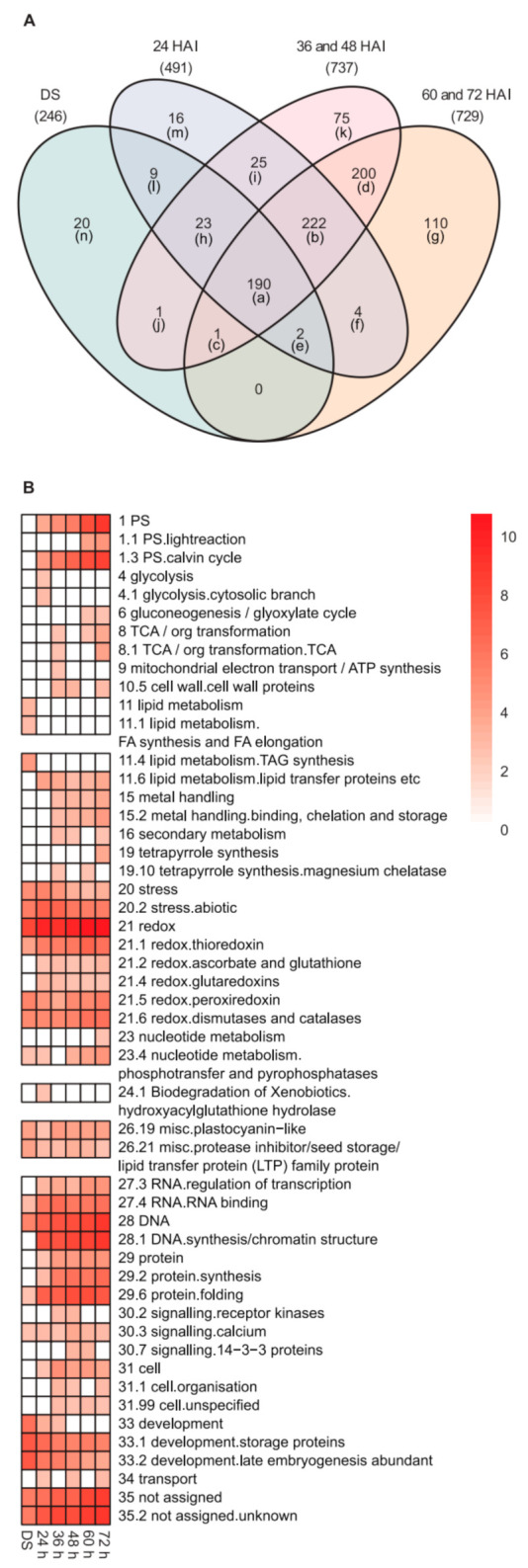
Heat-stable proteins in dry seeds (DS), germinating seeds and seedlings. (**A**) Venn diagram showing common and unique heat-stable proteins at the indicated time points after imbibition. (**B**) Functional enrichment of MapMan terms in the heat-stable proteome of DS, germinating seeds and seedlings. The white/red color gradient represents z-scores calculated from *p*-values (*p* < 0.01).

The heatmap of 898 heat-stable proteins (Supplemental Figure S3) revealed that most of the proteins had one directional change in abundance, i.e., either increased or decreased during germination and seedling establishment. Some clusters showed also a transient increase for 24 to 48 HAI. The number of detected proteins in each time point is shown in a Venn diagram using proteins from DS, 24 HAI, 36 together with 48 HAI and 60 together with 72 HAI data sets (Figure 4A, Supplemental Table S4). The total number of detected proteins is increasing over time. The two time points with the least number of common proteins were DS and 60 and 72 HAI (193 proteins). On the contrary, the highest common protein number was found when overlapping 36 and 48 HAI proteins and 60 and 72 HAI proteins (613 proteins). After excluding eight proteins, which were only present in either one of the combined time points (marked in yellow in Supplemental Table S4a), we were left with 182 common heat-stable proteins that were found throughout germination and early seedling development. Among them, CpHsc70-1 (AT4G24280) and LEA20 (AT2G40170) had the highest increase and decrease in abundance in 72 HAI seedlings compared to DS, respectively (Supplemental Table S3).

To explore the assignment of the 898 heat-stable proteins to main metabolic pathways and other processes we performed a functional enrichment analysis (Figure 4B, Supplementary Table S5) as statistical measure of whether a particular functional group (bin) of proteins contains more up- or down-regulated proteins than expected if all detected proteins would be equally distributed among all bins. We observed significant enrichment of 23 MapMan bins in DS, germinating seeds and seedlings. Nine bins were enriched in all observed time points; stress (20) with sub-bin abiotic stress (20.2), redox (21) with sub-bins thioredoxin (21.1), peroxiredoxin (21.5), dismutases and catalases (21.6), misc-related proteins including plastocyanin-like (26.19) and inhibitor/seed storage/lipid transfer protein (LTP) family protein (26.21), RNA binding (27.4), DNA (28), protein folding (29.6), signaling calcium (30.3), development-related proteins including storage protein (33.1) and late embryogenesis abundant proteins (33.2) and not assigned (35 and 35.2). Among these, redox, DNA and not assigned bins were highly enriched throughout the observation window. RNA binding (27.4) and protein folding (29.6), on the other hand, were highly enriched after 24 HAI.

Proteins from some bins and sub-bins were absent in DS but found in germinating seeds and young seedlings. These bins were PS (1) with sub-bin Calvin cycle (1.3), lipid transfer proteins (11.6), ascorbate and glutathione (21.2) and glutaredoxins (21.4), regulation of transcription (27.3), DNA synthesis/chromatin structure (28.1), protein (29) and its sub-bin synthesis (29.2) and cell (31). Enrichment of PS and Calvin cycle proteins clearly increased with HAI where it reached its peaks in young seedlings, coinciding with the transition from heterotrophic seeds to autotrophic seedlings. Most bins were enriched at more than one observed time point. However, proteins belonging to lipid metabolisms, (11) including FA synthesis and elongation (11.1) and TAG synthesis (11.4), were enriched only in DS while the enrichment of tetrapyrrole synthesis (19) and nucleotide (23) proteins was specific to seedlings 72 HAI.

Additionally, kinetic patterns within the heat-stable proteome were investigated for the 182 proteins, which were common among DS and all samples after imbibition using k-means clustering. Clustering revealed seven patterns of changes in protein abundance (Supplemental Figure S4A) which contained proteins assigned to 21 bins (Supplementary Figure S4B). The five bins with the highest proportion were: not assigned (17.6%), proteins (synthesis/post-translational modification/degradation/folding) (12.6%), redox (11.0%), development (9.90%) and stress (8.24%).

Among the clusters with a larger number of members (clusters 2, 3, 4 and 5), no single functional bin dominated. Excluding cluster 6 with only four members, the most common bins found across all clusters were stress (six clusters) and proteins (five clusters). Some bins were shared among clusters with the same protein abundance pattern. Heat-stable proteins that belonged to the PS, proteins and stress bins were found in all clusters with an overall increase in protein abundance after imbibition (clusters 1, 2 and 4). On the other hand, development was the common bin among clusters with a decrease in abundance (clusters 3, 6 and 7).

### 2.3. The Heat-Stable Proteome Exhibited an Equal Distribution of Intrinsically Ordered and Disordered Proteins

Since it is well-established that most LEA proteins are intrinsically disordered proteins [38] and that these proteins are heat-stable [49], we analyzed these sequences within the heat-stable proteome to predict disorder content in each protein using the MFDp2 online tool [50] (Table 1, Supplemental Table S3). Of the 898 heat-stable proteins, 701 amino acid sequences were retrieved from the UniProtKB/Swiss-Prot database (Supplemental Table S3). Various degrees of disorder content were found in the heat-stable proteins, from 0% disorder (12%) to 100% (10.8%). Hence, an almost equal fractioning of fully ordered and fully intrinsically disordered proteins (IDP) was observed. Proteins with a predicted disorder content between 0 and 10% comprised the biggest group in our data set (20.5%) followed by the group with a predicted disorder content of 10–20% (15.8%). The protein number tended to be smaller with increasing degree of disorder. In fact, proteins with at least 50% disorder content comprised only 21.8% of the whole data set, clearly indicating that disorder is not a prerequisite for heat stability. Likewise, small and large proteins were found in all disorder categories. The mean size of proteins was similar among most categories. The smallest and largest means were found in 70–80% and 90–100% disorder predicted protein, respectively (199.1 vs. 461.4 residues).

**Figure 5:**
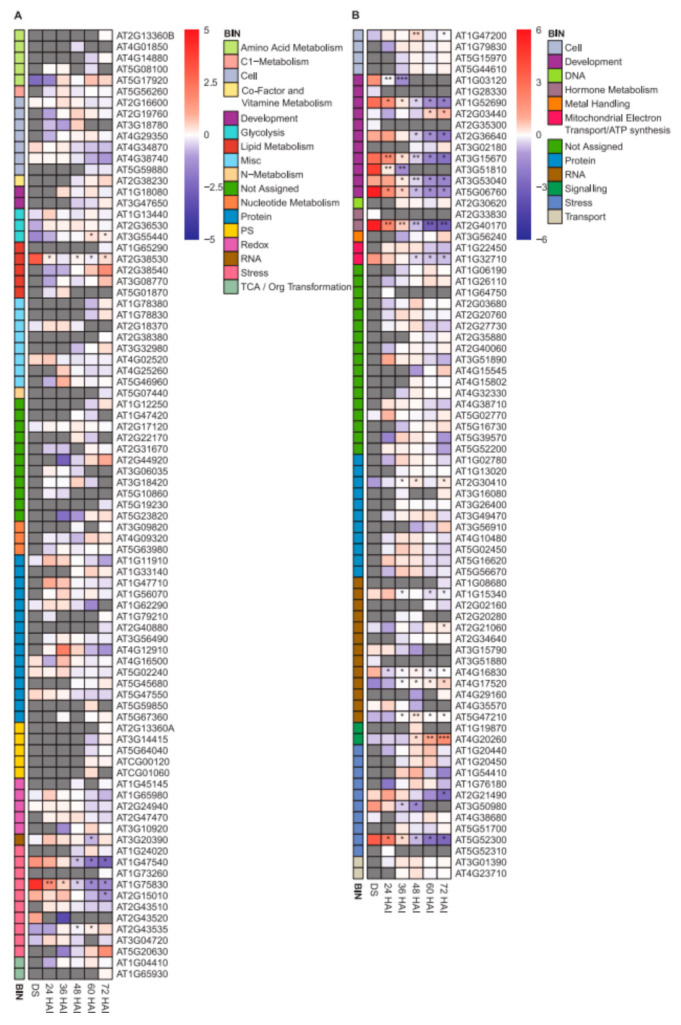
Abundance of proteins with (**A**) 0% and (**B**) 100% disorder content in the heat-stable proteome in dry seeds (DS), germinating seeds and seedlings. The color gradient represents the log_2_-median transformed means of mass spectral LFQ intensities (*n* = 3). Gray boxes represent missing values at the indicated time point. Levels of significance comparing protein abundance in germinating seeds and seedlings to DS (unpaired two-sided *t*-test) are indicated by asterisks (* *p*< 0.05; ** *p* < 0.01; *** *p* < 0.001). Proteins are assigned to MapMan bins, each bin is indicated by a unique color as shown. The transcript AT2G13360 mapped to two bins and was therefore marked with A and B. For additional information, see Supplemental Table S6.

To investigate if the grade of disorder is related to functional categories, the protein abundance for proteins with 0% and 100% disorder content was exemplarily assigned to MapMan bins using TAIR10 annotations [51] during the investigated germination time-course (Figure 5, Supplemental Table S6). While there was no distinct pattern found among fully folded proteins in terms of protein abundance among the bins identified, we could see some pattern among the fully disordered proteins. For example, there was an increasing abundance within 72 HAI for fully disordered proteins in the following bins: cell, protein, signaling and transport. The majority of these proteins was undetected in DS and early stages of germination, and therefore, a significance test to protein abundance in DS was often impossible due to missing values at this condition. Significant decrease in abundance of 100% disordered proteins within 72 HAI was found in the bins development and hormone metabolism.

### 2.4. LEA Protein Dynamics during Seed Germination

From the 898 heat-stable proteins in the data set, 29 LEA proteins in eight of the nine LEA Pfam groups [42] were identified (Supplemental Table S7). Only members of the Pfam LEA_3 group were not detected. The Pfam group with the largest number of members was LEA_4 (37.9%) followed by dehydrin (20.7%) and seed maturation protein (SMP) (10.3%). Out of 29 LEA proteins in the heat-stable proteome, 17 sequences were retrieved from the UniProtKB/Swiss-Prot database. The length of these LEA proteins ranged between 97 and 479 amino acids. Most of them (76.5%) were predicted by MFDp2 to have 100% disorder content (Supplemental Table S7). Only four of them were predicted with a smaller fraction of disorder content: LEA21 with 16.4%, LEA22 with 12.1%, LEA31 with 24% and LEA32 with 48.8%. 

To observe whether the high proportion of fully disordered LEA proteins is only found in the heat-stable proteome, we investigated sequences of LEA proteins which were reported to be seed-expressed [42] but were not detected in our data set Supplemental Table S8). We were able to retrieve 10 of these from the UniProtKB/Swiss-Prot database and found that 30% of them were predicted to be 100% disordered. The missing LEA protein with the lowest disorder content was LEA50 with 0%, followed by LEA1 (3.3%) and LEA27 (13.9%). 

A PCA of the LEA protein data set (Figure 1D) showed a clear separation between samples from different time points. Similar to the heat-stable proteome data set, PC1 explained 69.2% of the total variance and separated DS from all time points after imbibition, while PC2 (15.6% of the total variance) separated germinating seeds and young seedling samples.

**Figure 6:**
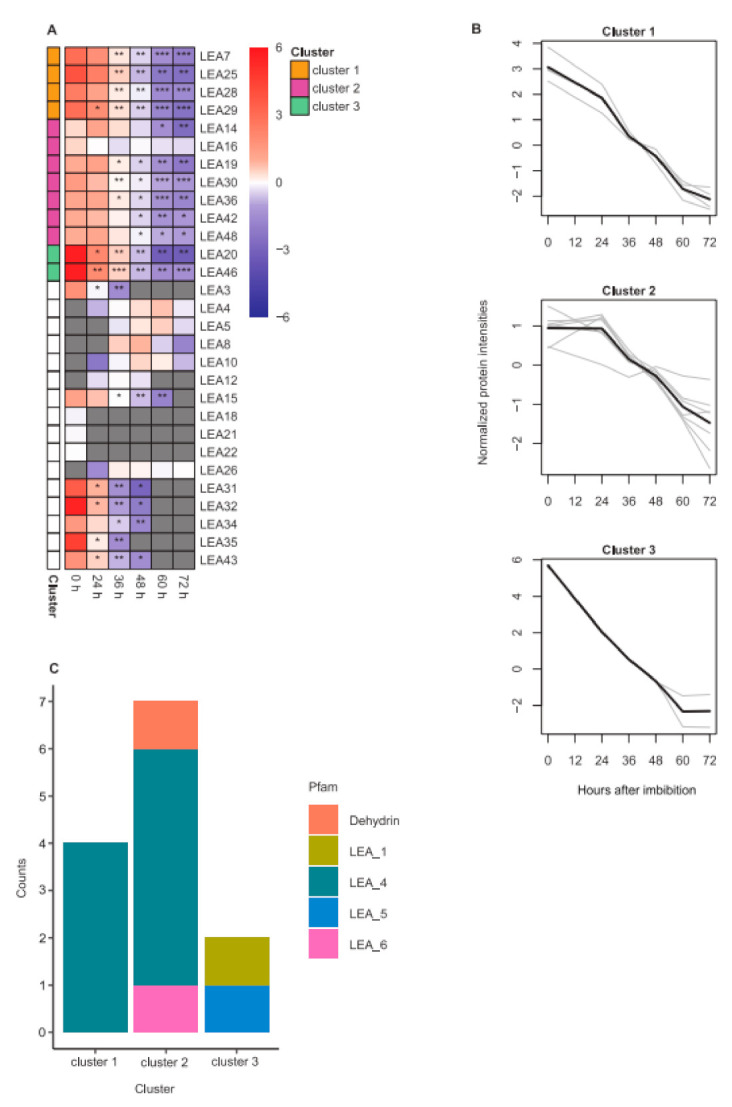
LEA protein abundance in dry seeds (DS), germinating seeds and seedlings and their kinetic patterns and Pfam distributions. (**A**) LEA protein abundance in DS and at different time points after imbibition. The color gradient represents the log_2_-median transformed means of mass spectral LFQ intensities (*n* = 3). Gray boxes represent missing values at the indicated time point. Levels of significance comparing LEA protein abundance in germinating seeds and seedlings to DS (unpaired two-sided *t*-test) are indicated by asterisks (* *p*< 0.05; ** *p* < 0.01; *** *p* < 0.001). LEA proteins are listed according to their LEA ID and corresponding clusters (cluster 1–3) as determined by k-means clustering. Each cluster is represented by a unique color. LEA proteins excluded from k-means clustering are depicted in white. (**B**) Patterns of LEA protein abundance as determined by k-means clustering. Gray lines represent individual proteins while black lines show the mean of each cluster. (**C**) Counts of LEA proteins in different Pfam groups in each cluster. Each Pfam group is represented by a unique color.

Most of the detected LEA proteins (79.3%) were found in DS (Figure 6A) and most of these showed a significant reduction in abundance after imbibition that became more pronounced with time. Some of these LEA proteins (34.5% and 37.9%) were no longer detecTable 60 and 72 HAI. Noticeably, three LEA proteins (LEA18 from LEA_1 and LEA21 and LEA22 from the AtM Pfam groups) were specific to DS. On the other hand, six LEA proteins (LEA4, LEA5, LEA8 and LEA10 from the dehydrin, LEA12 from the LEA_4 and LEA26 from the LEA_2 Pfam groups) became only detectable in germinating seeds from 24 or 36 HAI onwards. Thus, they were referred to as germination-specific. LEA12, however, was already undetectable again 60 HAI, indicating a very specific function during early germination.

To observe patterns of changes in LEA protein abundance, k-means clustering was applied to 13 LEA proteins found across all time points (Figure 6B). Although members of all clusters were reduced after imbibition, three clusters of LEA proteins from different Pfam groups with different kinetics were identified (Figure 6C). LEA_4 proteins were the most commonly found in the cluster data set with 69.2%. Proteins in cluster 1 showed a constant gradual decrease. Proteins in the largest cluster 2 (53.8% of cluster data set) showed a steady abundance up to 24 HAI, followed by a gradual decrease except for LEA16. Proteins in cluster 3 largely decreased in abundance up to 60 HAI with little further change over the last 12 h. LEA20 was the LEA protein with the largest reduction in abundance between DS and 72 HAI among all clustered proteins.

### 2.5. Transcript Abundance of Genes Encoding LEA Proteins

**Figure 7:**
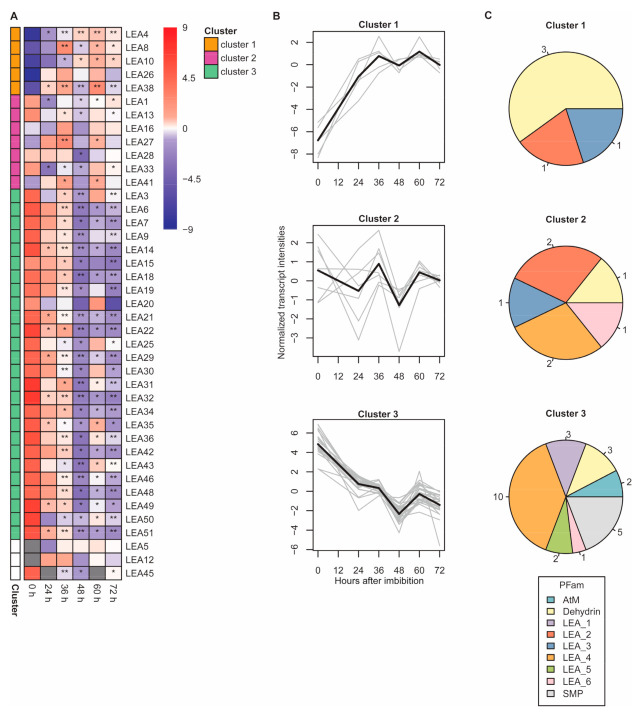
Transcript levels of genes encoding LEA proteins in dry seeds (DS), germinating seeds and seedlings after imbibition. (**A**) LEA transcripts at different time points after imbibition. The color gradient represents means of DW-normalized absolute transcript copy number in log_2_ with median normalization (*n* = 3). Gray boxes represent missing values at the indicated time point. Level of significance comparing LEA transcript abundance in germinating seeds and seedlings to DS (unpaired two-sided *t*-test) are indicated by asterisks (* *p* < 0.05; ** *p* < 0.01; *** *p* < 0.001). LEA transcripts are listed according to their LEA ID and corresponding clusters as determined by k-means clustering. Each cluster is represented by a unique color. LEA transcripts excluded from k-means clustering are depicted in white. (**B**) Patterns of LEA transcript abundance as determined by k-means clustering. Gray lines represent individual transcripts while black lines show the mean of each cluster. (**C**) Distribution of Pfam of LEA transcripts in each cluster. Each Pfam is represented by a unique color. Numbers in pie charts represent the numbers of Pfam members.

While we detected 29 LEA proteins in the heat-stable proteome, transcripts encoding 34 LEA proteins were previously detected by qRT-PCR in dry Arabidopsis seeds [42]. Here, we investigated the transcript levels of these LEA genes in DS and after imbibition along with genes encoding LEA proteins in the heat-stable proteome that were not among those 34. Therefore, we investigated in total 41 LEA genes. The abundance of LEA transcripts was expressed in absolute copy number of transcripts normalized to dry weight (DW) of samples used for RNA extraction (copy/gDW). Copy numbers of LEA transcripts were highest in DS, with a maximum average of 3.38 × 10^13^ copy/gDW for LEA20, and lowest in seedlings 72 HAI, with a minimum average of 9.47 × 10^6^ copy/gDW for LEA50 (see Supplemental Table S9 for the complete data set). We detected transcripts for LEA1, LEA6, LEA9, LEA13, LEA27, LEA33, LEA38, LEA41, LEA45, LEA49, LEA50 and LEA51 (for LEA27, LEA38 and LEA41 with higher abundance at 36 and 60 DAI) even though no corresponding protein was detected (Figure 7A). 

A PCA of the LEA transcript data (Figure 1E) showed a separation between DS and all time points after imbibition by PC1, which explained 75% of the total variance. However, unlike for the LEA protein data (Figure 1D), there was no clear separation of the time points after imbibition by PC2, suggesting that the kinetics of LEA transcript appearance were less defined than those of the proteins.

K-means clustering showed three different transcript abundance patterns of the observed LEA transcripts (Figure 7B). Due to presence of missing values, LEA5, LEA12 and LEA45 were excluded from k-means clustering. All LEA Pfam groups were represented across the three clusters (Figure 7C). Cluster 1 comprised LEA transcripts that increased in abundance after imbibition. The increase was gradual up to 36 HAI, and then transcript levels generally remained constant. The transcript of LEA38 was the only one in cluster 1, which did not encode germination-specific LEA proteins as described above. Cluster 2 contained LEA transcripts with similar abundance over time and only small fluctuations. Cluster 3 contained transcripts with reduced abundance after imbibition with the lowest abundance at 48 HAI. This cluster represented the majority of the observed LEA transcripts (68.4% of cluster data set) and was dominated by LEA_4 transcripts (38.5%). Cluster 3 was the most diverse cluster consisting of LEA transcripts from 7 Pfam groups, only missing members from LEA_2 and LEA_3 groups, which were both detected in cluster 1 and cluster 2 (Figure 7C). Cluster 3 was the only cluster with LEA transcripts from AtM, LEA_1, LEA_5 and SMP Pfam groups.

Since we determined both LEA protein and transcript abundances, we were able to investigate the correlation between mRNA and protein levels. We only investigated the correlations between 23 LEA proteins and their transcripts when detected in at least four of the six observed time points. Significant correlations were found in nine of these twenty-three cases, which came from four different Pfam groups of LEA_1, LEA_4, dehydrin and SMP (Table 2). The highest correlation between the abundance of a protein and the corresponding transcript was found for LEA32 from SMP Pfam group. Of the nine significantly correlated pairs of proteins and transcripts, only the germination-specific dehydrins LEA4 and LEA10 were upregulated, while the other seven were downregulated during germination. A delay in protein accumulation following the transcript accumulation was observed for some LEA proteins. For example, for LEA10, a delay of at least 24 h has been found between the increase in transcript and protein detection.

### 2.6. Protein–Metabolite Network Analyses Reveal a LEA Cluster with High Connectivity

As the majority of LEA proteins common for all time points showed a fast decrease during seed germination and seedling establishment, we were interested in other proteins and metabolites following the same pattern to identify highly correlated protein or protein–metabolite networks associated with seed germination. With this approach, we wanted to integrate omics data for an interpretable network for the discovery of molecular interactions previously not discovered for heat-stable proteins under these conditions. A protein–metabolite network analysis was performed, including all heat stable proteins (182) together with all metabolites (70) and amino acids (20) common for all six time points. For reduction in edges, a cut off (r ≥ 0.99 and a *p*-value < 0.05) was chosen (Supplemental Table S10). The identified network included 91 proteins (including 11 LEA proteins), 42 metabolites and 13 amino acids with a varying range of correlations and a high connectivity level (Supplemental Figure S5). LEA proteins (colored in purple) had multiple relationships to each other and revealed positive connections building a small sub-cluster with LEA7, LEA19, LEA25, LEA28, LEA29, LEA30, LEA36, LEA42 and LEA48. Among them, LEA7, LEA25, LEA28, LEA29 and LEA30 were identified as network hubs with a high level of inter-connectivity represented by eight to eleven edges. These LEA proteins were closely correlated with several heat-stable proteins as the seed storage proteins SESA1, a bi-functional inhibitor/lipid-transfer protein/seed storage 2S albumin superfamily protein (AT4G30880), the ABA-inducible dehydration-responsive protein RD29B/LTI65, the eukaryotic elongation factor 5a (ELF5a) and a protein of unknown function (AT1G16850). The large subunit of Rubisco (RBCL) displayed several negative correlations with LEA proteins (LEA25, LEA29, LEA42) and other members in this sub-network. Only three metabolites were involved in this cluster, namely phosphoric acid, 1,5-anhydro-sorbitol and an unknown analyte (A167004).

This large LEA cluster was connected to another large cluster including mainly metabolites of the central metabolism, most of the amino acids, sugar phosphates, *myo*-inositol-phosphate and phosphoglyceric acid via a positive correlation to LEA20. LEA20 together with LEA46 was more closely correlated with several other heat-stable proteins and metabolites. For LEA20, correlations were found with the seed storage protein SESA4, the Low-Molecular-Weight Cysteine-Rich 67 (LCR67) involved in pathogen response and an unknown protein (AT3G12960). LEA46 showed also positive connections to LCR67, a cytochrome c oxidase (AT1G32710) and a serine protease inhibitor (AT2G38870), possibly involved in biotic stress response. LEA46 showed in contrast to the other LEA proteins a high number of negative correlations with metabolites and amino acids, including mannose-6-phosphate, myo-inositol-phosphate, isoleucine, glutamine and threonine.

## 3. Discussion

Seed germination is a complex process, which is accompanied by comprehensive changes and a concerted action of molecular players represented by gene expression, protein abundance and metabolite levels. Dry seeds with a quiescent metabolic state undergo a transition to proliferative metabolic state for plant propagation [22]. Molecular omics changes during seed germination in Arabidopsis were studied before but not much attention was directed to variations of the heat-stable proteome including intrinsically disordered proteins and especially changes in LEA proteins and their correlation network with metabolic changes.

### 3.1. Degradation of Storage Compounds to Central Metabolites Contributes to the Transition from Heterotrophic to Autotrophic Growth

Fifty-six known and forty-three unknown metabolites were identified over a time frame of 72 HAI, which is comparable to other publications on Arabidopsis [8,11], wheat [16] and tomato seeds [17]. Following a previous classification [11], metabolite profiles showed major shifts between three developmental stages: (1) the dry state, (2) initial stages of germination (24 HAI and 36 HAI) and (3) greening and fully opened cotyledons (48 HAI to 72 HAI). Similar to our findings, a PCA clearly distinguished metabolite profiles of wheat embryo and endosperm after 24 h of germination from the profiles at 0 h [16]. Six metabolites, namely fumaric acid, galactinol, sucrose, raffinose, 2,5-dihydroxybenzaldehyde and 3,4-dihydroxybenzoic acid, revealed high levels in DS and were decreased during seed germination and seedling establishment, underlining their role as storage reserves and protective substances for dry seeds [30]. Sucrose or raffinose family oligosaccharides (RFOs) are the most abundant non-reducing sugars in dry mature seeds [36] and serve as storage reserves during germination, resulting in their depletion for energy production and heterotrophic growth before the transition to autotrophic growth [10]. A continuous decrease in sucrose and galactinol as precursor of RFO during seed to seedling transition in Arabidopsis was reported previously [10,11]. Sucrose was additionally suggested as a signaling metabolite during seed germination as sucrose levels showed correlations with 129 transcripts including high numbers of sucrose-responsive genes [10].

Galactinol was described as marker for seed longevity in Brassicaceae and tomato [52]. An overexpression of galactinol synthase of *Cicer arietinum* in Arabidopsis led to higher seed longevity by reducing reactive oxygen substance (ROS) accumulation [53]. On the other hand, galactinol synthase 1 was reported as negative regulator of seed germination [54]. For fumaric acid, an increase during seed maturation was shown, especially during desiccation, resulting in high levels in dry seeds [8].

The two metabolites 2,5-dihydroxybenzaldehyde and 3,4-dihydroxybenzoic acid, which were present in DS, 24 HAI and 36 HAI, have been, to our knowledge, not described in seed germination studies before. 3,4-dihydroxybenzoic acid (protocatechuic acid) was widely detected in cereals and legumes and other species [55,56]. It affects growth of tobacco callus and organs, with small concentrations inducing root growth while high concentrations inhibited shoot and root growth [57]. Furthermore, positive correlations between antioxidant enzyme activity and 3,4-dihydroxybenzoic acid were found in Miscanthus [58].

Most of the other identified metabolites, sugars and sugar phosphates, amino acids and most organic acids were increased with proceeding germination following the activation of biosynthetic processes for growth and development. They were found previously within 24 h germination or till opening of the cotyledons [8,11].

Consistent with other studies, intermediates of the TCA cycle were rather reduced during germination whereas metabolites involved in glycolysis, such as sucrose-derived sugar-phosphates, were increased, supporting the finding that the main energy source for seed development is glycolysis [8,11,16].

A release of amino acids anticipating radicle protrusion and seedling growth was described to start at 24 HAI [22]. Canonical Correlation Analysis (CCA) of metabolite changes during 6 h of seed germination in tomato revealed close correlations of characteristic combinatorial changes in a larger set of metabolites but not individual metabolites with germination parameters. This larger set included metabolites of central metabolic pathways as sucrose, glucose, fructose, metabolites of the TCA cycle as fumarate and succinate and in addition glycerol-3-phosphate, the sugar alcohols *myo*-inositol and galactinol and several amino acids [17]. Altogether, the metabolic status of the here-described experiment was similar to previously published studies, which allows us to compare our data on the heat-stable proteome with previous publications.

### 3.2. Thermal Stability of Proteins Is Related to Biological Processes

Whereas metabolic shifts during seed germination were previously investigated, less is known about the heat-stable proteome including low abundant proteins at different stages of seed germination. A total of 898 heat-stable proteins were identified in seeds over 72 h of germination in our study, which was the equivalent of around 30% of 2967 proteins with high melting temperature (T_m_) or no melting point (nonmelters) in Arabidopsis [59]. Shifts in protein abundance of the heat-stable proteome during seed germination over time differed from that of metabolites. Proteome samples from all time points were clearly separated from each other (except 60 HAI and 72 HAI), whereas metabolite profiles of 24 HAI and 36 HAI were closely clustered.

Heat-stable proteins were enriched in the following bins at all time points: stress, redox, RNA binding, DNA, protein folding and development-related proteins including storage and late embryogenesis abundant proteins. The functional bin photosynthesis with the sub-bin Calvin cycle was highly enriched from 24 HAI. Previously, heat-stable proteins were described to be enriched for ribosomal, RNA-binding and protein biosynthesis processes [60], and functional enrichment analysis of heat-stable Arabidopsis proteins by thermal protein profiling (TPP) pointed to bins related to protein folding, carbon fixation and the proteasome [61], thus revealing an overlap with our findings. Interestingly, an enrichment of proteins involved in carbon fixation was observed including RuBisCo subunits [61], as we also discovered in our data set. 

For human cell lines, it was suggested that differences in thermal stability might reflect differences in the activity of biological processes [59]. It was also hypothesized that heat-induced cell damage is mainly caused by the denaturation of a relatively small set of functionally essential “hub” proteins [60]. Following our functional enrichment analysis, this hypothesis can be confirmed as heat-stable proteins were enriched in biological processes important for ongoing seed germination and greening of the cotyledons as photosynthesis, Calvin cycle, abiotic stress and redox reactions. 

A comparison of the here-discovered heat-stable proteome of 898 proteins of the Arabidopsis accession Col-0 with 256 proteins previously identified with two-dimensional gel-electrophoresis in the accession Landsberg erecta (Ler) showed an overlap of 149 proteins, revealing a heat-stable percentage of 58% in the Ler data set [22]. The overlap included a large amount of heat-stable proteins involved in biological processes, e.g., several seed storage proteins as cruciferins (CRA1, AT5G44120; CRU2, AT1G03880; CRU3, AT4G28520) belonging to the most abundant cruciferins in DS and serving as nitrogen and amino acid source for seedling development. Additionally, abundant 12S cruciferins were hypothesized to be predominant ROS scavengers in seeds being highly exposed to oxidative stress [22]. 

In the early germination phases, the de novo synthesis of key enzymes of the glyoxylate cycle, such as isocitrate lyase (AT3G21720), aconitase 3 (AT2G05710) and malate synthase (AT5G03860), was reported [22], pointing to lipid remobilization. All of them were also included in the heat-stable proteome of Col-0. Malate dehydrogenase (AT1G04410), converting malate into oxaloacetate for amino acid synthesis pathways or conversion to phosphoenolpyruvate by phosphoenolpyruvate carboxykinase 1 (AT4G37870), was also found in both studies. 

Furthermore, heat-stable proteins common in both studies are involved in antioxidant defense and detoxification: iron/manganese superoxide dismutase (AT3G56350), 1-cysteine peroxiredoxin (AT1G48130), catalase 2 (AT4G35090), thioredoxin-dependent peroxidase 1 (AT1G65980), ferritin 2 (AT3G11050) and monodehydroascorbate reductase 6 (AT1G63940). Generation of ROS takes place during water uptake, which might have negative effects on proteins and other cell components [62]. In dry pea seeds, superoxide dismutase, catalase, ascorbate peroxidase, dehydroascorbate reductase and glutathione reductase were detected as being involved in radical scavenging during imbibition [62].

The restart of the cellular metabolism during germination involves elements of the cytoskeleton, including actin and tubulin, and consequently, tubulin 3 (AT5G62700), actin 2 (AT3G18780) and actin 7 (AT5G09810), which were identified in both data sets and are heat stable. Finally, proteins involved in stress response as heat shock proteins (HSP70, AT3G12580; HSC70.5, AT5G09590; HSP60.2, AT2G33210) [22] overlapped between the studies, underlying the thermal stability of proteins involved in important biological processes during germination. The classification of heat-stable proteins into main metabolic processes and defense responses and the enrichment in functional bins representing biological processes point to mobilization of storage proteins and lipids, an activated heterotrophic and later autotrophic metabolism and several stress response mechanisms during seed germination. 

### 3.3. An Equal Distribution of Fully Ordered and Fully Intrinsically Disordered Proteins Characterized a Part of the Heat-Stable Proteome of Germinating Seeds

The loose structure of intrinsically disordered proteins (IDP), e.g., LEA proteins, contributes to their heat-stability when globular proteins aggregate [63]. Often, IDPs are enriched after heating, e.g., when comparing heat-stable proteins with the whole proteome of soybean [64]. An increased content of IDPs was also reported in the heat-stable protein fraction of imbibed radicles of *Medicago truncatula* [41]. In a study compiling an atlas of the thermal stability of 48,000 proteins across 13 species ranging from archaea to humans, nonmelters were most strongly enriched in disordered regions [59].

Nevertheless, a surprisingly high percentage of low disorder content was found for the heat-stable proteome in our study, independent of protein length. In contrast, a study estimating the relative content of intrinsic protein disorder in 96 plant proteomes reported an inverse relationship between the degree of intrinsic protein disorder and protein length [65]. A high intrinsic disorder does not seem to be the main requirement for the heat stability of proteins. A more complex relationship between disorder and thermal protein stability may exist [59]. Recently, features as molecular weight, hydrophobicity, charged versus polar (CvP) bias and protein helix and sheet composition were shown to be highly correlated with thermal stability of Arabidopsis proteins. In addition, cytoplasmic protein concentration, interactions of small molecules and cellular localization were suggested to have an effect [59,61], whereas protein abundance was controversially discussed as either not a good predictor of thermal stability or important for stability [59]. Thermal stability of proteins can be additionally changed by phosphorylation [66] or the redox state [67].

While no functional category was favored in proteins with 0% disorder content, 100% disordered proteins of the heat-stable proteome during seed germination and seedling establishment were mainly assigned to the functional MapMan bins development, DNA, protein, RNA and stress. The disorder-related functions of plant proteins were previously related to stress tolerance, transcription regulation, cell cycle regulation, molecular chaperones and developmental regulation [64]. In addition, IDPs were mainly involved in the GO terms regulation of nucleus activities, regulation of metabolic processes, response to biotic and abiotic stimuli and signaling, depicting an overlap with our results even that functional bins were differently classified [65]. When investigating proteins from 13 species, highly unstructured proteins were enriched in nuclear and phosphorylated proteins [59]. On the other hand, nonmelters with low disorder were enriched in transmembrane-domain-containing proteins, proteins with extracellular domains, glycosylated proteins, secreted proteins with signal peptides and proteins containing disulfide bonds [59].

### 3.4. Dehydrins Were Identified as Seed Germination-Specific LEA Proteins

Within the heat-stable proteome 29 LEA, proteins were detected with the vast majority of them having 100% disorder. An analysis of the heat-stable proteome of imbibed radicles or seeds of *Medicago truncatula* aiming to investigate proteins linked to desiccation tolerance identified 11 to 16 LEA proteins [41,47]. 

The ABA-induced accumulation of LEA proteins in parallel to the accumulation of non-reducing sugars during seed maturation provide resistance to desiccation [36,68]. Seed rehydration imposes severe stress, including leakage of solutes and temporary membrane and organelle damage from free radicals. Consequently, synthesis of proteins and compounds which limit and repair cell damage is prevalently needed [6]. With proceeding water uptake during seed germination and the loss of desiccation tolerance, the abundance of most LEA proteins declines sharply [24,68]. 

The 10 LEA proteins previously described as seed-expressed in Arabidopsis [42] were not detected in the heat-stable proteome of germinating seeds (LEA1, 6, 27, 33, 37, 38, 41, 49, 50, 51), even if 100% disordered (LEA6, 33, 51). Most LEA proteins were detected in DS, including three LEA, specific to DS and decreased during germination. LEA proteins were decreasing and partially remaining during germination in Arabidopsis [18] and pea [69] with a protective function postulated for the remaining proteins.

Only a small number of LEA proteins appeared at later time points. LEA12 was only present from 24 HAI to 48 HAI and was previously described as bud specific [42]. Five other LEA proteins were only present from 36 HAI and were defined as germination specific (LEA4-synonym COR47, LEA5-ERD10/LTI45/LTI29, LEA8-HIRD11, LEA10-ERD14, LEA26), most of them belonging to the class of dehydrins and predicted to be 100% disordered. Three of the five germination-specific LEA proteins were previously described as stress induced [42], which suggests the possibility of contributing to stress tolerance during the development of seedlings. 

The protein abundance of LEA4 and LEA10 was highly correlated with the related transcript levels, whereas for the other germination-specific LEAs (LEA5, 8, 26), transcripts were present earlier compared to proteins, suggesting the possibility of a fast de-novo synthesis during seed germination. 

Dehydrins contain highly conserved stretches of 7-17 residues that are repetitively scattered in their sequences, the K-, S- and Y-rich segments, and accumulate during seed maturation and in response to abiotic stress as cold, dehydration, osmotic stress or ABA [70]. Some of them are phosphorylated in response to stress and are then capable of calcium binding (e.g., ERD14). Dehydrins are able to bind to phospholipids and thereby to modulate membrane properties [39]. In most studies, their abundance is reduced during seed germination (for a review, see [68]). Nevertheless, these proteins were also shown to be involved in seed germination and development [71]. LEA5/ERD10 is important for completion of seed development as *erd10* mutants revealed abnormal shape and reduced germination [71]. Results on the expression of *ERD10* in DS are controversial, from no expression (our results and [72]) to detection in DS [71]. 

LEA4/COR47, LEA5/ERD10 and LEA10/ERD14 were previously identified as members of protein complexes. Homodimeric and heterodimeric interactions were verified for LEA4/COR47 and LEA5/ERD10 in the cytosol of tobacco cells by bimolecular fluorescence complementation, but also heterodimeric associations between LEA51/RAB18 or PIP2B, and these dehydrins took place in the cytosol [73,74]. For LEA4/COR47, the transcription factor WRKY63, involved in seedling establishment and growth, was identified as an upstream regulator, pointing to a possible role in seedling development [75].

LEA8/HIRD11 has RNA binding activity, containing an RNA G-quadruplex structure (RG4) in its 3′UTR, which folds with guanine-rich sequences [76]. These complexes play a role in post-transcriptional regulation of gene expression, plant development and growth modulation [76]. An *HIRD11* mutant showed reduced root growth and retarded shoot growth.

LEA26, a germination-specific LEA beside LEA12 not belonging to the class of dehydrins, was shown to function as a protector (chaperon) of cellular components from stress, as this protein prevented the inactivation of the enzyme lactate dehydrogenase (LDH) during freezing and stabilized membrane structures [77].

All of these germination-specific, stress-related dehydrins in addition to their specific functions might protect germinating seeds from environmental stresses after the reduction in osmoprotective substances such as raffinose and other protecting LEA proteins. As an example, they were able to provide cold stress resistance to highly watered seed [68].

### 3.5. LEA Proteins Build a Highly Correlated Cluster in the Seed Germination Specific Common Protein–Metabolite Network

The construction of a protein–metabolite network helped to develop a deeper understanding of their pattern during seed germination, even though only heat-stable proteins and metabolites were included common for all investigated time points. The discovery of a tight LEA protein cluster among all common proteins during germination is mainly based on a common decrease in abundance of all LEA proteins at the same time during imbibition, but is still mentionable, as only very little proteins follow the same pattern. For that reason, the following discussion of this network focuses mainly on the roles of the specific LEA proteins in DS and very early germination rather than for seedling establishment. Several LEA proteins were identified as network hubs, including LEA7, LEA25, LEA28, LEA29 and LEA30, all belonging to the Pfam group LEA_4.

As a high number of metabolites in the network were unknown, not many conclusions could be drawn regarding common protein–metabolite accumulation patterns that might reflect interactions. Nevertheless, the LEA proteins of the LEA cluster were separated from almost all other metabolites. All of these LEA proteins play important roles for seed protection in the dry state also represented by connections to seed storage proteins and a seed storage 2S albumin superfamily protein. LEA7 in leaves had a protective function on enzymes during freezing and drying [78] and its structural transitions upon drying were modulated by the presence of membranes [79]. A connection of several LEA proteins to the ABA-inducible dehydration-responsive protein RD29B/LTI65 might be related to the induction of LEA proteins by ABA [80,81]. In *nced2569* (9-cis epoxycarotenoid dioxygenase), a quadruple mutant with ABA deficiency, expression of genes encoding LEA28, LEA29, LEA48 and LEA42 was strongly downregulated together with 1-Cys peroxiredoxin1 (Per1), all involved in our LEA cluster [81]. The negative connectivity to RBCL was also interesting, which might point to a protective role of LEA proteins as chaperones for this protein before the induction of photosynthesis during development.

Only LEA20 and LEA46 showed connectivity to a cluster, including metabolites and amino acids of the central metabolism and were only weakly connected to the LEA cluster. LEA20 from Pfam group LEA_5, also named EM6, is induced by ABA and salt [42] and mutant analysis revealed that LEA20 is required for normal seed development in Arabidopsis [82]. LEA20 has a role in water binding and loss during embryo maturation, as mature seed from these mutants lose more water than wild-type seeds during air drying and show an accelerated acquisition of desiccation tolerance [83].

The hub LEA46 (Pfam group LEA_1) with high connectivity might have a protective function, e.g., for enzymes as cytochrome-c-oxidase involved in respiration. The overexpression of LEA46 conferred drought tolerance to severe stress [84]. This might be due to the chaperone-like activity after conformational transition from disorder to alpha-helix folding [85]. Negative correlation of abundance patterns of LEA46 with that of isoleucine, glutamine and threonine might point to a mechanism switching from protection of enzymes during the dry state to securing biosynthesis of crucial metabolites during the developmental stages. Threonine serves as precursor of isoleucine, important for root development, as isoleucine deficiency impairs root development in Arabidopsis [86]. Furthermore, all three amino acids might serve as precursor for glutathione, important as antioxidants and for detoxification [23].

A demonstration of the functional relevance of the identified putative interactions will be necessary in the future to fully understand the importance of the putative protein–metabolite network during seed germination.

## 4. Materials and Methods

### 4.1. Plant Materials and Experimental Conditions

*Arabidopsis thaliana* (accession Col-0) plants were grown on soil in a climate chamber with 20 °C/60% relative humidity (RH) during the day and 6 °C/70% RH during the night in a 14 h/10 h light/dark cycle with of 180 μmol m^−2^ s^−1^ light intensity. After a week, plants were moved to a phytotron with an 8 h light period at a light intensity of 200 μmol m^−2^ s^−1^, 20 °C, 60% RH and 16 °C during the 16 h night at 75% RH for another week before pricking. After pricking, plants were kept at these conditions for two additional weeks to gain more leaf biomass before being transferred to long-day conditions in a greenhouse (16 h light at 21 °C, 8 h night at 19 °C and 50% RH during day and night) until seed harvest. Plants were bagged after siliques matured to minimize seed loss. After bagging, plants were left to dry for two weeks in the greenhouse. Seeds were collected in the bags and stored in the dark at 15 °C/15% RH for three weeks before they were used for experiments.

Samples for metabolomics, proteomics and transcript analysis were taken from dry seeds (DS), germinating seeds and young seedlings (24, 36, 48, 60 and 72 HAI). Seeds (except for DS) (75 mg of DS per replicate, 3–5 replicates per time point) were sterilized with 70% ethanol for 2 min, followed by 9% bleach solution containing 0.02% Triton X-100 for 17 min and five washings with sterile water. Seeds were then sown on blue germinating paper (grade 190, 300 g/m^2^, Sartorius, Göttingen, Germany) on agar plates containing half-strength MS medium without sucrose. Seeds on plates were stratified at 4 °C in the dark for 24 h before transfer to a growth chamber (22 °C, 16 h light period). Germinating seeds and seedlings were collected 24, 36, 48, 60 and 72 HAI by flash freezing with liquid nitrogen in a mortar. After homogenizing dry seed samples using mixer mill MM200 (Retsch, Haan, Germany) and germinating seeds and seedlings with mortar and pestle, fresh weight (FW) of the samples was determined before extractions for further analysis. Dry weight (DW) of samples was determined from 60 mg FW of plant material after drying overnight at 4 °C in a freeze dryer.

### 4.2. Metabolite Profiling

Aliquots of small polar metabolites were prepared from five biological replicates of each time point using 60 mg of ground fresh material. The extraction used water/methanol: chloroform with lipid partitioning as previously described [87]. ^13^C_6_-sorbitol (Sigma-Aldrich, Taufkirchen, Germany) was added during the extraction and used as internal standard. The analysis, using 160 µL from the upper polar phase, dried overnight in a vacuum concentrator, was carried out using gas chromatography after derivatization with 40 µL methoxymation reagent in pyridine and 80 µL silylation-mix for tri-methyl-silylation in split 1/30 and splitless mode with 1 µL injection volume coupled to electron impact ionization-time of flight-mass spectrometry (GC/EI-TOF-MS) [87]. ChromaTOF software (LECO Instrumente GmbH, Mönchengladbach, Germany) was used to process the acquired chromatograms. Identification of metabolites was conducted using TagFinder [88], the NIST08 software (http://chemdata.nist.gov/dokuwiki/doku.php?id=start (accessed on 28 January 2020)) and the mass spectral and retention time index reference collection of the Golm Metabolome Database [89,90,91,92]. Statistical analysis was based on mass spectral intensities were normalized to dry weight and ^13^C_6_-sorbitol.

The mass spectrometry metabolomics data have been deposited into the MetaboLights database [93] with the dataset identifier MTBLS2980.

### 4.3. Amino Acid Analysis

Dried 160 µL of the upper polar phase extracted for metabolite profiling were used for the analysis. Four biological replicates per time point were resuspended in 65 µL of 0.1 M HCl and centrifuged at 14,000 rpm at 4 °C for 15 min. The measurement of amino acids was carried out using reverse HPLC as previously described [94] with small adjustments in LC gradient. Amino acid content was calculated based on the peak area of the mass fragments normalized to DW of the sample.

### 4.4. Proteomic Analysis of Heat-Stable Proteins

Extraction of total soluble protein and extraction of the heat-stable proteome (95 °C, 10 min) were performed as described [41] from three biological replicates from each time point. Protein concentration was determined using Bradford reagent (B6916, Sigma-Aldrich, Taufkirchen, Germany)/(Bio-Rad Laboratories, Hercules, CA, USA) and bovine serum albumin as a standard. Then, 100 µg of protein per sample was mixed with 100 µL of 8 M urea in 10 mM Tris-HCl, pH 8.0. Samples were loaded on filter columns (Microcon-30 kDa Centrifugal Filter Unit with Ultracel-30 membrane). Columns were washed with 8 M urea, 10 mM Tris-HCl, pH 8.0. Next, proteins were reduced using 10 mM DTT in 8 M urea, 10 mM Tris-HCl, pH 8.0 and alkylated using 27 mM iodoacetamide in the same buffer. Samples were mixed at 600 rpm for 1 min and then incubated without mixing for an additional 5 min. Subsequently, columns were washed using 8 M urea, 10 mM Tris-HCl, pH 8.0 and centrifuged. Proteins in the columns were digested for 14 h using mass spec grade Trypsin/Lys-C mix (Promega, Madison, WI, USA). The resulting peptides were desalted on C18 SepPack columns (Teknokroma, Barcelona, Spain) and eluted with 800 µL 60% acetonitrile, 0.1% trifluoroacetic acid, dried in the speed vacuum concentrator and stored at −80 °C. Measurements were performed on a Q Exactive HF high-resolution mass spectrometer (Thermo Scientific, Waltham, MA, USA) coupled to an ACQUITY UPLC M-Class System (Waters, Milford, MA, USA). Peptide samples were loaded onto an Acclaim PepMap RSLC reversed-phase column (75 μm inner diameter, 25 cm length, 2 µm bead size (Thermo Scientific, Waltham, MA, USA) at a flow rate of 0.4 μL min^−1^ in 3% (*v*/*v*) acetonitrile, 0.1% (*v*/*v*) formic acid. Peptides were eluted by an acetonitrile gradient from 3% to 80% (*v/v*) over 120 min at a flow rate of 0.5 μL/min. Peptide ions were detected in a full scan from mass-to-charge ratio 300 to 1600 at resolution of 60,000. MS/MS scans were performed for the top ten MS scans with the highest MS signal (ddMS2 resolution of 15,000, AGC target 3e5, isolation width mass-to-charge ratio 1.4 *m*/*z*, relative collision energy 30). Peptides for which MS/MS spectra had been recorded were excluded from further MS/MS scans for 20 s.

Raw data were processed using MaxQuant software [95] and the *A. thaliana* TAIR10 annotations (Arabidopsis TAIR database Version 10, The Arabidopsis Information Resource, www.Arabidopsis.org (accessed on 27 February 2019)) in combination with the search engine Andromeda [96]. The settings for MaxQuant analysis were set as follows: trypsin and lysine selected as digesting enzyme, two missed cleavages allowed, fixed modification was set to carbamidomethylation (cysteine) and oxidation of methionine was set as variable modification. Spectra were also searched against a decoy database of the *A. thaliana* proteome and results were filtered to obtain a FDR below 1% on the protein level. The “label-free quantification” and “match between runs” options were selected. A minimum peptide length of six amino acids was used. The quantification was performed for proteins with a minimum of one unique and one razor peptide. Known contaminants, such as keratins, and proteins, which were identified with only one unique peptide, were removed from further analysis. Statistical analysis was based on mass spectral label-free quantification (LFQ) intensities.

The mass spectrometry proteomics data have been deposited into the ProteomeXchange Consortium via the PRIDE [97] partner repository with the dataset identifier PXD027546.

### 4.5. qRT-PCR Analysis

Total RNA was extracted from three biological replicates of each time point. The extraction was performed as described [98], with minor modifications. During the first step of RNA extraction, six artificial RNAs from the ArrayControl RNA Spikes kit (Ambion, Austin, TX, USA) were added to the samples as internal standard to allow calculation of the absolute number of transcript copies [99,100]. The number of copies of the different RNA spikes per extract were as follows: Spike 1 (6.08 × 10^9^), Spike 2 (1.52 × 10^9^), Spike 3 (4.56 × 10^8^), Spike 4 (1.14 × 10^8^), Spike 5 (2.94 × 10^7^) and Spike 8 (7.60 × 10^5^). RNA concentration and quality were assessed using a Nanodrop One UV/VIS spectrophotometer (Thermo Scientific, Waltham, MA, USA). Genomic DNA in the RNA samples were removed using RapidOut DNA removal kit (Thermo Scientific, Waltham, MA, USA). Absence of DNA was confirmed by qPCR using primers designed for an intron sequence of *rbcS* (Supplemental Table S11) [101]. All qPCR reactions used 2× SYBR Green Master Mix Reagent (Applied Biosystems, San Francisco, CA, USA) in optical 384-well plates using 7900HT Fast Real-Time PCR System (Thermo Scientific, Waltham, MA, USA). cDNA was synthesized using PrimeScript RT reagent kit with the use of Oligo dT primers (Takara Bio, Kusatsu, Japan). To check the quality of cDNA, qPCR was performed using primers designed for 5′ and 3′ ends of *GAPDH* (Supplemental Table S11) [99]. All samples were found with good quality, as |Ct5′–Ct3′| of *GAPDH* was between 0–1.

To observe the dynamics of LEA transcripts at different HAI, we used primers specific for 41 LEA genes and the six RNA spikes. This included 34 LEA genes that were previously identified as expressed in dry seeds [42] and eight additional genes encoding LEA proteins identified by our proteomics analysis. Sequences of all primers targeting LEA transcripts except LEA35 and RNA spikes have been published previously [42,100] and are listed in Supplemental Table S11. The absolute copy number of each LEA transcript was calculated based on the linear correlation of the CT values and log_10_ copy number of RNA spikes in all samples. Extrapolation based on the linear correlation was carried out for transcripts with a copy number of more than 6.08 × 10^9^. Absolute copy numbers were normalized to sample DW prior to statistical analysis.

### 4.6. Statistical and Other Analyses

All statistical analyses were performed with RStudio version 1.2.5033 [102]. Prior to performing Principal Component Analysis (PCA) and unpaired two-sided *t*-test, data imputation was performed if a metabolite was detected in three out of five and a protein or transcript was detected in two out of three replicates per time point. The imputation was performed using half the minimum value of either DW-normalized mass-spectral intensities (for metabolites), LFQ intensity (for heat-stable proteins) or DW-normalized absolute copy number (for LEA transcripts). Analytes with more than 40% missing value at a time point were not considered for further evaluation. All data were log_2_-transformed and median-normalized for statistical analyses.

In addition, for the heat-stable protein data set, prediction of disorder content of the proteins was carried out using the MFDp2 online tool [50]. The protein sequences of 701 proteins from our data set were available on the UniProtKB/Swiss-Prot database [103]. Unique and common proteins in each time point were shown in a Venn diagram from “VennDiagram” package version 1.6.20. Moreover, functional enrichment of heat-stable proteins was performed according to bins assigned by MapMan using TAIR10 annotations [51]. Enrichment of MapMan bins in the heat-stable proteome was analyzed based on LFQ intensities, which were added with 1 after imputation, thus allowing log_10_ transformation of proteins with previous intensities of 0 without affecting the intensities of other proteins. CorTo v.1.0.3 (http://www.usadellab.org/cms/index.php?page=corto (accessed on 6 January 2021) was used to analyze the enrichment by conducting Fisher’s exact test followed by the Benjamini and Hochberg correction with of *p* value of 0.01 as a threshold. Z-scores were calculated from *p* values using the inverse normal cumulative distribution function.

PCA was performed using the “pcaMethods” package version 1.74.0 [104]. For PCA, missing values were replaced with 0 and mean values were Pareto-scaled and mean-centered using the probabilistic method. Abundance of analytes from DS to 72 HAI were shown in heat maps which illustrated the log_2_-median transformed mean of each analyte at each observed time point. Only the missing values in the heat map showing the abundance of all heat-stable proteins were replaced with 0 to allow clustering using Euclidean clustering on the heat map, otherwise, the missing values remained. Clustering for other heat maps was performed based on the k-means clustering approach by excluding metabolites, proteins or transcripts with missing values from k-means clustering. Clusters were further characterized based on classes of metabolites, protein bins as assigned by MapMan [51] and Pfam groups of LEA proteins [42]. Unpaired two-sided *t*-testwas performed to test for significant differences between abundance at DS and subsequent time points. Pearson correlation tests were performed between the abundance of LEA proteins and their corresponding transcripts for those with protein and transcript detection from at least four of the six investigated time points. All *p* value were adjusted using the Benjamini–Hochberg procedure [105] for false discovery rate correction. 

### 4.7. Network Analysis of Common Metabolites, Amino Acids, Heat-Stable Proteins

Mean of log_2_-transformed and median-normalized data of metabolites, amino acids and heat-stable proteins which could be detected in DS and samples from all observed time points after imbibition were included in a network analysis using Cytoscape version 3.7.2 [106] with the threshold cut-off of Benjamini–Hochberg adjusted *p* < 0.05 and Pearson correlation r ≥ |0.99|, as described previously [107]. Network statistics of the undirected protein–metabolite interaction network revealed a power law with R^2^ = 0.782, indicating that the network is scale-free. 

## Figures and Tables

**Table 1:** Heat-stable proteins with different predicted disorder content. A total of 701 protein sequences were retrieved from UniProtKB/Swiss-Prot. The prediction was performed by MFDp2. The table shows protein counts and other information (Min = minimum, Max = maximum, Mean of length of residues in each group of predicted disorder content).

Predicted Disorder Content	Counts	Min	Max	Mean
(%)		(Residues)		
0	84	80	843	255.6
0–10	144	56	990	342.3
10–20	111	82	974	310.5
20–30	97	69	976	348.9
30–40	62	87	987	341
40–50	50	72	953	309.8
50–60	27	98	724	294.2
60–70	18	93	772	303.4
70–80	9	129	367	199.1
80–90	7	111	577	277
90–100	16	131	891	461.4
100	76	62	956	284.9

**Table 2:** Pearson correlation between the abundance of LEA proteins and their corresponding transcripts. LEA proteins with missing means mass spectral LFQ intensities in more than two observed time points were excluded from analysis. Levels of significance of the correlations are indicated by asterisks (** *p* < 0.01; *** *p* < 0.001).

LEA ID	Pfam (Hundertmark and Hincha, 2008)	Pearson Correlation	*p* value
LEA4	dehydrin	0.703 **	0.010
LEA5	dehydrin	−0.091	0.839
LEA7	LEA_4	0.669 **	0.009
LEA8	dehydrin	0.052	0.873
LEA10	dehydrin	0.789 **	0.005
LEA14	dehydrin	0.212	0.577
LEA15	LEA_6	0.581	0.061
LEA16	LEA_6	−0.169	0.664
LEA19	LEA_4	0.461	0.131
LEA20	LEA_5	0.250	0.507
LEA25	LEA_4	0.409	0.191
LEA26	LEA_2	0.050	0.873
LEA28	LEA_4	0.070	0.839
LEA29	LEA_4	0.695 **	0.006
LEA30	LEA_4	0.297	0.393
LEA31	SMP	0.839 **	0.005
LEA32	SMP	0.934 ***	0.0003
LEA34	dehydrin	0.813 **	0.006
LEA36	LEA_4	0.127	0.715
LEA42	LEA_4	0.346	0.310
LEA43	LEA_4	0.777 **	0.009
LEA46	LEA_1	0.824 ***	0.0004
LEA48	LEA_4	0.243	0.507

## Data Availability

All data are available in the Supplementary Materials. In addition, the proteomics data are available at ProteomeXchange via the accession number PXD027546. The metabolomics data are available at MetaboLights via MTBLS2980.

## Supplementary Materials

The following are available online at https://www.mdpi.com/article/10.3390/ijms22158172/s1. Figure S1: Seeds and seedlings collected after imbibition. Figure S2: Relative metabolite abundance in dry seeds (DS), germinating seeds and seedlings and their kinetic patterns and class distributions. Figure S3: Relative abundance of heat-stable proteins in dry seeds (DS), germinating seeds and seedlings collected at the indicated time points after imbibition. Figure S4: Kinetic patterns of 182 heat-stable proteins common in dry seeds (DS), germinating seeds and seedlings at indicated time points after imbibition. Figure S5: Network analysis of heat-stable proteins with metabolites and amino acids common in dry seeds (DS), germinating seeds and seedlings. Table S1: Mass-spectral intensities of metabolites normalized to the internal standard ^13^C_6_-sorbitol and dry weigh in dry seeds (DS), germinating seeds and seedlings at indicated time points after imbibition. Table S2: Amino acid content normalized to dry weight (nmol/gDW) in dry seeds (DS), germinating seeds and seedlings at indicated time points after imbibition. Table S3: LFQ intensities, annotation and disorder content of heat-stable proteins in dry seeds, germinating seeds and seedlings at indicated time points after imbibition. Table S4: Proteins in different section of the Venn diagram (Figure 4A) with their TAIR10 annotation. Table S5: Functional enrichment of MapMan bins of heat-stable proteins found in dry seeds, germinating seeds and seedlings at indicated time points after imbibition. Table S6: LFQ intensities, annotation and disorder content of fully folded (0% disorder) and fully disordered (100% disorder) heat-stable proteins in dry seeds, germinating seeds and seedlings at indicated time points after imbibition (see Figure 5). Table S7: LFQ intensities and disorder content of LEA proteins found in heat-stable proteome in dry seeds, germinating seeds and seedlings at indicated time points after imbibition. Table S8: Disorder content of seed-expressed LEA proteins not found in the heat-stable proteome of dry seeds, germinating seeds and seedlings. Table S9: Copy number of LEA transcript normalized to dry weight (copy/gDW) in dry seeds, germinating seeds and seedlings at the indicated time points after imbibition. Table S10: Pearson correlations and Benjamini–Hochberg adjusted *p* value of analytes included in the network. Table S11: List of primers used for RNA quality check and qRT-PCR.
