# Supplementary material for: Characterization of the Heat-Stable Proteome during Seed Germination in Arabidopsis with Special Focus on LEA Proteins
Source: Int J Mol Sci. 2021 Jul 29;22(15):8172. doi: 10.3390/ijms22158172 (PMC8347141; doi:10.3390/ijms22158172)
Supplement: Supplementary file 1 [file ijms-22-08172-s001.zip › ijms-1272535-suplementary figures.pdf]

Ginsawaeng et al., Supplemental Figure S1

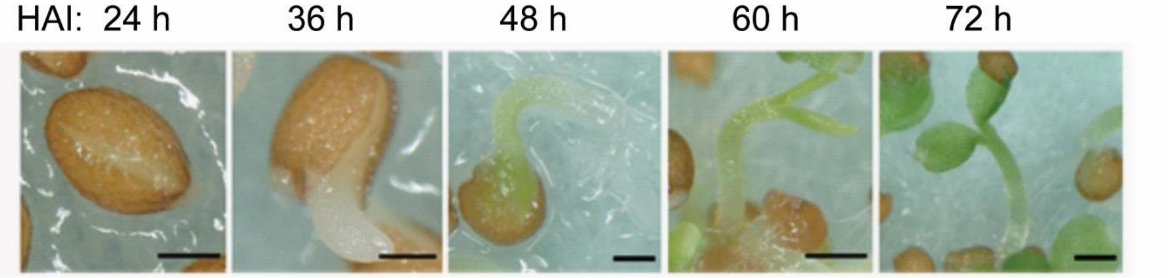

Supplemental Figure S1: Seeds and seedlings collected after imbibition. Dry seeds were sterilized and placed on germination paper on half-strength MS agar plates without sugar. Seeds were stratified at 4°C for 24 h before they were transferred to a growth chamber. Pictures were taken at the indicated hours after imbibition (HAI). The scale bar denotes 1 mm.

Ginsawaeng et al., Supplemental Figure S2

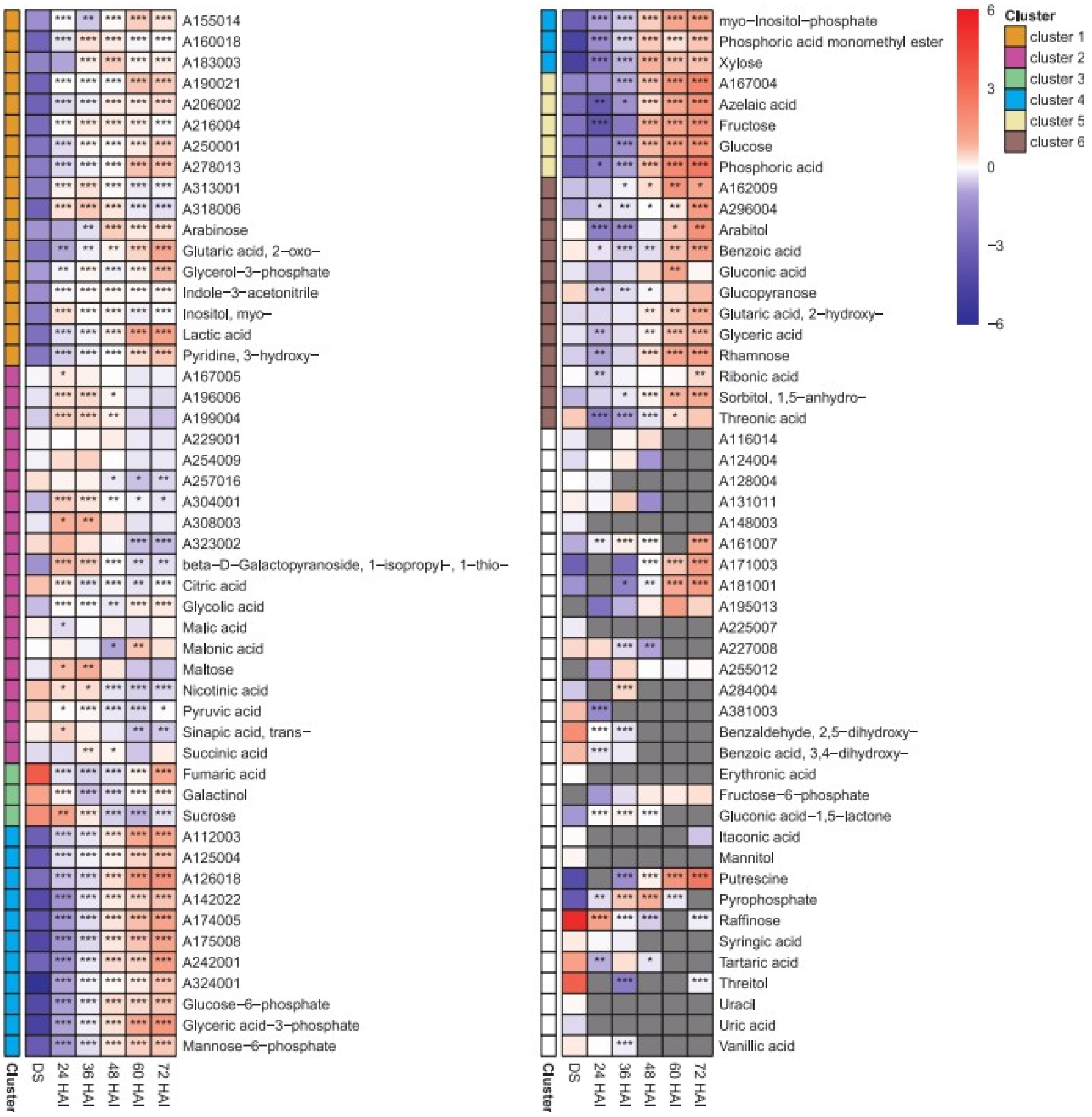

Supplemental Figure S2: Relative metabolite abundance in dry seeds (DS), germinating seeds and seedlings and their kinetic patterns and class distributions. (**A**) Abundance of metabolites in DS, germinating seeds and seedlings collected at the indicated time points after imbibition. The colour gradient represents means of log_2-median transformed DW-normalized mass-spectral intensities (n = 5). Grey boxes represent missing values at the indicated time point. Level of significance comparing the metabolite abundance in germinating seeds and seedlings to DS (Student's t-test) are indicated by asterisks (* P < 0.05; ** P < 0.01; *** P < 0.001). Metabolites are listed alphabetically within their clusters (cluster 1-6) as determined by k-means clustering. Each cluster is indicated by a unique colour shown to the left. Metabolites excluded from k-means due to missing values clustering are color-coded in white.

Ginsawaeng et al., Supplemental Figure S3

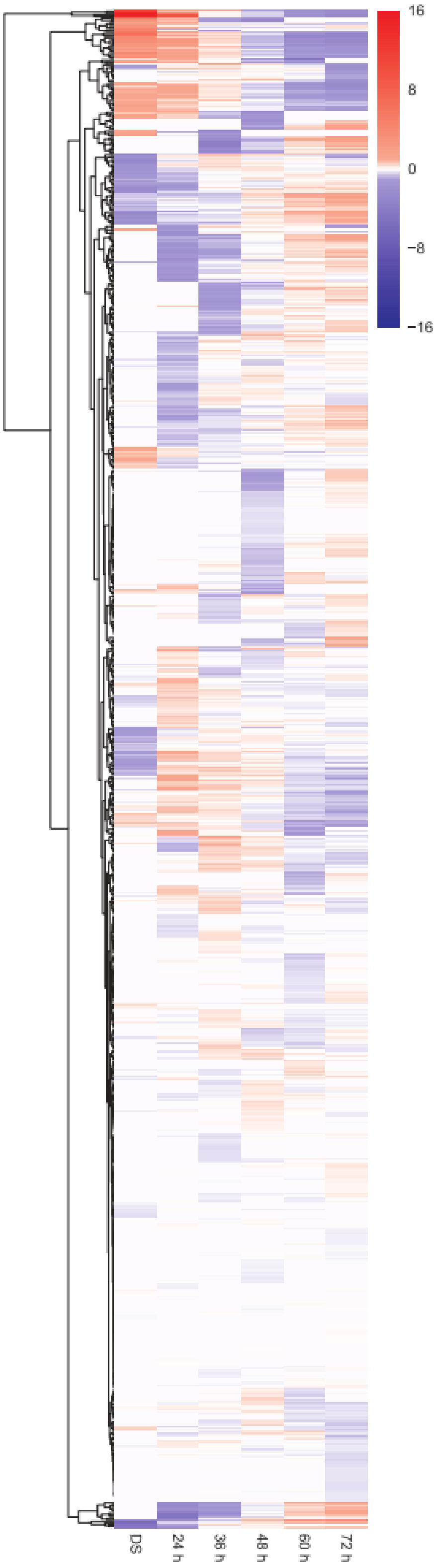

Supplemental Figure S3: Relative abundance of heat-stable proteins in dry seeds (DS), germinating seeds and seedlings collected at the indicated time points after imbibition. The blue/white/red color gradient represents the log_2-median transformed means of mass spectral LFQ intensities (n = 3).

Ginsawaeng et al., Supplemental Figure S4

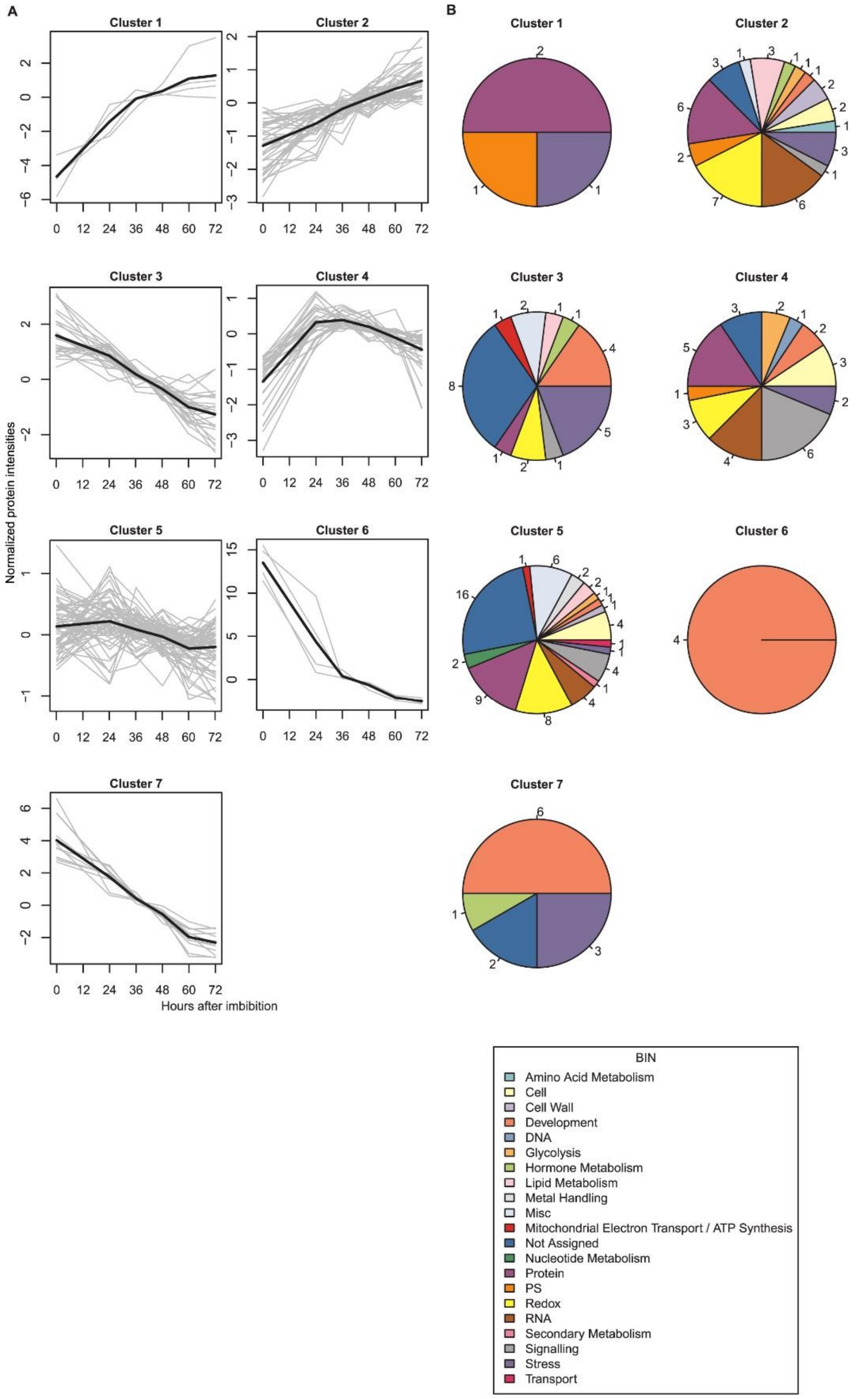

Supplemental Figure S4: Kinetic patterns of 182 heat-stable proteins common in dry seeds (DS), germinating seeds and seedlings at indicated time points after imbibition. (**A**) Patterns of protein abundance as determined by k-means clustering (cluster 1-7). Grey lines represent individual proteins while black lines show the representative pattern of each cluster. (**B**) Distribution of functional bins of proteins in each cluster as assigned by MapMan. Each bin is represented by a unique color as shown. Numbers around the pie charts represent the numbers of bin members.

Ginsawaeng et al., Supplemental Figure S5

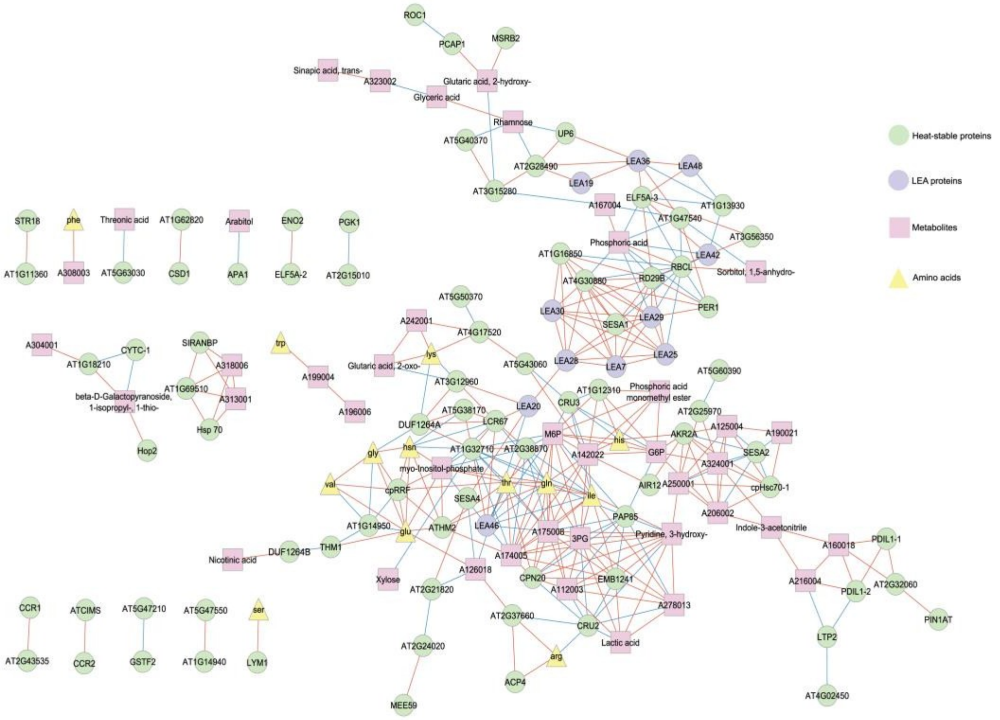

Supplemental Figure S5: Network analysis of heat-stable proteins with metabolites and amino acids common in dry seeds (DS), germinating seeds and seedlings. Red and blue arrows show edges which represent positive and negative Pearson correlations, respectively (r ≥ 0.99, Benjamini-Hochberg corrected p-value <0.05). Each analyte is represented by a unique colour and symbol as shown. 3PG = Glyceric acid-3-phosphate, G6P = Glucose-6-phosphate, M6P = Mannose-6-phosphate, AT1G03680 52 = THM1, AT1G03880 = CRU2, AT1G05510 = DUF1264A, AT1G07140 = SIRANBP, AT1G08830 53 = CSD1, AT1G11910 = APA1, AT1G16730 = UP6, AT1G21750 = PDIL1-1, AT1G21880 = LYM1, 54 AT1G22840 = CYTC-1, AT1G26630 = ELF5A-2, AT1G48130 = PER1, AT1G62740 = Hop2, AT1G69410 = ELF5A-3, AT1G75830 = LCR67, AT1G77510 = PDIL1-2, AT1G79920 = Hsp 70, AT2G18040 = PIN1AT, AT2G21660 = CCR2, AT2G36530 = ENO2, AT2G38530 = LTP2, AT3G07390 = AIR12, AT3G12780 = PGK1, AT3G22640 = PAP85, AT3G63190 = cpRRF, AT4G02520 = GSTF2, AT4G03520 = ATHM2, AT4G20260 = PCAP1, AT4G21860 = MSRB2, AT4G24280 = cpHsc70-1, AT4G25050 = ACP4, AT4G27140 = SESA1, AT4G27150 = SESA2, AT4G27170 = SESA4, AT4G28520 = CRU3, AT4G35450 = AKR2A, AT4G37300 = MEE59, AT4G38740 = ROC1, AT4G39260 = CCR1, AT5G17710 = EMB1241, AT5G17920 = ATCIMS, AT5G20720 = CPN20, AT5G45690 = DUF1264B, AT5G52300 = RD29B, AT5G66170 = STR18, ATCG00490 = RBCL.
